# Multi-omics analysis identifies GAA as an independent poor prognostic biomarker and candidate therapeutic target in angioimmunoblastic T-cell lymphoma

**DOI:** 10.3389/fimmu.2026.1862168

**Published:** 2026-08-11

**Authors:** Yanfei Liu, Yunfei Shi, Yang Zhao, Haojie Wang, Lan Mi, Meng Wu, Yuqin Song, Jun Zhu, Yan Xie

**Affiliations:** 1 Key Laboratory of Carcinogenesis and Translational Research (Ministry of Education), Department of Lymphoma, Peking University Cancer Hospital & Institute, Beijing, China; 2 Key Laboratory of Carcinogenesis and Translational Research (Ministry of Education/Beijing), Comprehensive Clinical Trial Ward, Peking University Cancer Hospital & Institute, Beijing, China; 3 Key Laboratory of Carcinogenesis and Translational Research (Ministry of Education/Beijing), Department of Pathology, Peking University Cancer Hospital & Institute, Beijing, China; 4 Technology Innovation Center of Mass Spectrometry for State Market Regulation, Center for Advanced Measurement Science, National Institute of Metrology, Beijing, China

**Keywords:** angioimmunoblastic T cell lymphoma, CD8+ T cell infiltration, lysosomal alpha-glucosidase, metabolic reprogramming, prognosis

## Abstract

**Background:**

Angioimmunoblastic T-cell lymphoma (AITL) is an aggressive subtype of peripheral T-cell lymphoma with poor clinical outcomes and limited therapeutic options. The contribution of metabolic reprogramming and immune microenvironmental alterations to AITL progression remains insufficiently defined.

**Methods:**

We conducted integrative transcriptomic and proteomic analyses of AITL samples compared with reactive lymphoid hyperplasia to identify molecules associated with treatment response and prognosis. Immune infiltration and pathway enrichment analyses were performed, and findings were validated in independent GEO cohorts (GSE19069 and GSE58445). Key protein expression was confirmed by immunohistochemistry and multiplex immunofluorescence.

**Results:**

Differential expression analyses revealed that lysosomal alpha-glucosidase (GAA), a key enzyme involved in glycogen metabolism, was significantly upregulated at both the RNA and protein levels in patients who failed to respond to standard chemotherapy. Elevated GAA expression was associated with inferior overall survival in multivariate analysis. Immunohistochemistry staining and multiplex immunofluorescence further confirmed the spatial expression pattern of GAA in AITL tissues. Immune deconvolution and pathway enrichment analyses suggested that GAA-high tumors exhibited increased CD8^+^ T-cell infiltration accompanied by transcriptional features of T-cell exhaustion, as well as transcriptionally inferred metabolic alterations characterized by enhanced glycolysis-related signatures and reduced oxidative phosphorylation-related signatures. These findings were further validated in an independent AITL cohort from the GEO database.

**Conclusions:**

Our study identifies GAA as a candidate biomarker associated with adverse clinical outcomes, immune microenvironmental features, and metabolic pathway alterations in AITL, highlighting glycogen metabolism as a previously underexplored biological axis and a potential therapeutic vulnerability warranting further functional investigation.

## Graphical Abstract

**Figure:**
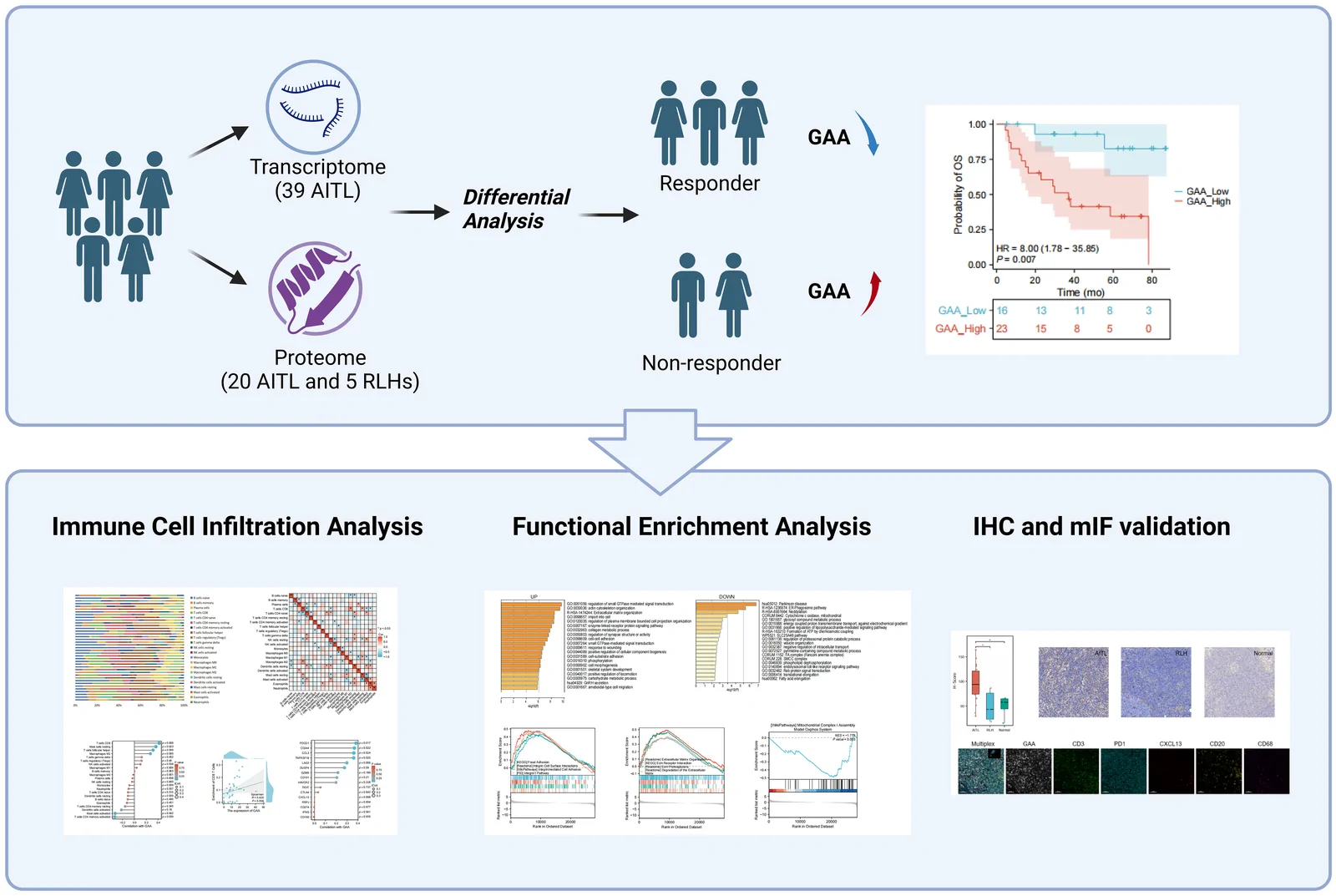

## 1 Background

Angioimmunoblastic T-cell lymphoma (AITL) is a rare and aggressive subtype of peripheral T-cell lymphoma that is clinically characterized by systemic symptoms, including fever, generalized lymphadenopathy, and autoimmune manifestations. Despite advances in the understanding of its pathogenesis, AITL remains associated with poor clinical outcomes, with most patients diagnosed at advanced stages and reported 5-year overall survival (OS) rates ranging from 32% to 44% (1–4). Current first-line treatment strategies are largely based on conventional chemotherapy regimens such as CHOP (cyclophosphamide, doxorubicin, vincristine, and prednisone), which have shown limited efficacy in improving long-term survival, particularly in relapsed or refractory disease (5, 6). Although several novel agents, including histone deacetylase inhibitors, EZH2 inhibitors, and CD30-targeted monoclonal antibodies, have demonstrated clinical activity in early-phase trials, their broader application remains limited by patient heterogeneity, acquired drug resistance, and the lack of reliable predictive biomarkers (7). Accordingly, there is a continued need to identify molecular determinants that underlie disease progression and therapeutic resistance in AITL.

Previous studies have extensively characterized the genomic and transcriptomic landscape of AITL. Recurrent mutations in genes such as RHOA, TET2, DNMT3A, and IDH2 have provided important insights into disease pathogenesis and contributed to the molecular classification of AITL (8, 9). Gene expression profiling has further shown that B-cell–associated signatures are linked to favorable clinical outcomes, whereas signatures related to monocytic differentiation, CD8^+^ T-cell cytotoxicity, and activation of the p53 pathway are associated with inferior survival (10). While these findings have substantially advanced the understanding of AITL biology, their translation into clinically actionable targets has been limited. In addition, the interaction between malignant T cells and the immune microenvironment remains incompletely understood, particularly with respect to the role of CD8^+^ T cells, which appear to exert context-dependent and potentially dysfunctional effects in AITL. Recent evidence suggests that metabolic reprogramming may influence both tumor behavior and immune cell function within the tumor microenvironment (11, 12); however, the metabolic features of AITL and their clinical relevance have not been systematically investigated.

In the present study, we performed integrated transcriptomic and proteomic profiling of AITL specimens with the aim of identifying disease-associated molecules with prognostic relevance. Using reactive lymphoid hyperplasia (RLH) as a control, we identified differentially expressed proteins associated with treatment response and found that lysosomal alpha-glucosidase (GAA), a key enzyme involved in glycogen metabolism, was consistently overexpressed at both the RNA and protein levels in patients who did not respond to standard chemotherapy. Elevated GAA expression was found to be associated with inferior overall survival in multivariate analysis. We additionally examined the association between GAA expression, immune cell infiltration, and related biological pathways, and validated these findings in an independent AITL cohort from the Gene Expression Omnibus (GEO) database. Together, these results indicate that GAA expression correlates with metabolic pathway signatures, immune microenvironmental features, and clinical outcome in AITL, supporting its role as a candidate prognostic biomarker and putative therapeutic vulnerability warranting further functional investigation.

## 2 Materials and methods

### 2.1 Patient samples

We retrospectively identified 39 newly diagnosed patients with AITL who had available tumor resection specimens and were treated in the Department of Lymphoma at Peking University Cancer Hospital between August 2014 and August 2022. Patients were included if they met the following criteria (1): a pathological diagnosis of AITL confirmed by experienced hematopathologists at our institution; (2) availability of archived tumor resection specimens; and (3) complete baseline clinical data. Patients who were lost to follow-up were excluded. In addition, five cases of RLH diagnosed during the same period were included as controls. For independent validation, transcriptomic data from two publicly available AITL cohorts (GSE19069 and GSE58445; n = 52) were retrieved from the GEO database (https://www.ncbi.nlm.nih.gov/gds/).

### 2.2 RNA sample preparation and sequencing

Total RNA was extracted from 39 formalin-fixed paraffin-embedded (FFPE) tumor samples using the miRNeasy FFPE Kit (Qiagen), according to the manufacturer’s protocol. RNA quality and concentration were assessed using an Agilent Bioanalyzer 2100 and a Qubit 3.0 Fluorometer, respectively. Ribosomal RNA was depleted using the KAPA Stranded RNA-seq Kit with RiboErase (HMR), and sequencing libraries were constructed following the manufacturer’s instructions. Libraries were sequenced in paired-end mode (150 bp) on an Illumina HiSeq 4000 platform. Raw sequencing reads were subjected to quality control using Trimmomatic (v0.33) and subsequently aligned to the reference genome using STAR (v2.5.3a). Gene-level expression quantification was performed with RSEM (v1.3.0).

### 2.3 Protein sample preparation and mass spectrometry-based proteomic analysis

Deparaffinized FFPE tissue sections were processed for protein extraction using 300 mM Tris-HCl containing 50% acetonitrile, followed by sequential water-bath sonication and heating. Proteins were reduced, alkylated, and digested with trypsin at an enzyme-to-protein ratio of 1:10 at 37 °C for 20 h. Peptides were acidified, centrifuged, and desalted using SDB-RPS StageTips. After washing and elution, peptides were vacuum-dried and stored at −80 °C until further analysis. Peptide separation was performed on a homemade C18 column (30 cm length, 1.9 μm particle size) using a 135-min gradient at a flow rate of 600 nL/min on an EASY-nLC 1200 system coupled to an Orbitrap Fusion Lumos mass spectrometer. Data were acquired in data-independent acquisition (DIA) mode, with MS1 and MS2 resolutions set to 120,000 and 30,000, respectively. DIA raw files were analyzed using Spectronaut (v14.9.201124) with the directDIA workflow. Digestion was specified as trypsin/P, dynamic mass tolerance calibration was applied, and retention times were aligned using the Biognosys iRT kit. A mutated decoy strategy was used to control false discovery rates. Protein and peptide identification and quantification were performed using default parameters with minor adjustments.

### 2.4 Bioinformatic analysis

The overall proportion of missing data was 13.8%, and missing values were imputed using the minimum observed value for each protein. Differential gene and protein expression analyses were performed using the limma package in R. TPM expression data were log2-transformed prior to analysis, and the ORR group was used as the reference group for all comparisons. Genes or proteins with |log_2_(fold change)| > 0.58 and a P-value < 0.1 were considered differentially expressed. Immune cell infiltration was estimated using the CIBERSORT algorithm (v1.03) with the LM22 signature matrix via the CIBERSORTx web portal. Functional enrichment analysis of differentially expressed genes was performed using the Metascape online platform (http://metascape.org) (13).

### 2.5 Immunohistochemistry

FFPE tissue sections (4 μm) from AITL samples were deparaffinized and subjected to heat-induced antigen retrieval using Tris–EDTA buffer (pH 9.0). Endogenous peroxidase activity was blocked with 3% hydrogen peroxide. Sections were incubated overnight at 4 °C with a rabbit polyclonal anti-GAA antibody (1:50 dilution; ET7110-77, Huabio), followed by incubation with an HRP-conjugated goat anti-rabbit IgG secondary antibody. Immunoreactivity was visualized using diaminobenzidine (DAB) and counterstained with hematoxylin. Images were acquired using a Nikon E100 microscope.

### 2.6 Multiplex immunofluorescence

Multiplex immunofluorescence (mIF) staining was performed with 7-color fluorescence immunohistochemistry kit (PhenoVision Bio Co., Itd) following the company manual instruction. In short, tissue sections went through deparaffinization, rehydration, and were transferred into citrate buffer pH6.0 for antigenic repair. The sections were then washed and incubated with H2O2 and blocking reagents for 10 minutes, respectively. Primary antibody (panel 1:PD1–PVB480, CD3–PVB520, CD20–PVB570, CD68–PVB620, CXCL13–PVB690, GAA–PVB780) was applied on each section for 30 min at RT (**Supplementary Table 1**). Bound primary antibodies were detected using PVB anti-Rb/Mm-HRP detection followed by PVB tyramide signal amplification fluorophore (PVB 480, PVB 520, PVB 570, PVB 620, PVB690, PVB780) for 10 min. The slides were counterstained with 4′,6-diamidino-2-phenylindole (PhenoVision Bio Co., Itd) and cover-slipped.

### 2.7 Image acquisition and data mining

The slides were scanned using the PhenoImager HT system (Akoya Biosciences) at 20× magnification. Image analysis was performed using the PhenoVision mIF AI analysis system (PhenoVision Bio Co., Ltd), trained from the Oncotopix Discovery system (Visiopharm), version 4.5.6.5 (Hoersholm, Denmark). For data analysis, the built-in AI algorithm was used to establish an application capable of identifying individual cells based on DAPI staining and recognizing cell phenotypes according to intracellular protein fluorescence signals. The region of interest (ROI) was defined as the entire tissue section, with large areas of adipose tissue excluded from analysis. Cell segmentation was performed using a U-Net–based recognition algorithm to identify valid cells, and cell fragments with an area < 8 μm² were excluded from analysis. Fluorescence intensity thresholds for marker positivity were defined as follows: PD1 (50), CD3 (20), CD20 (30), CD68 (10), CXCL13 (21), and GAA (50). The quantities of various cell phenotypes within the tissue were quantified, including but not limited to CD3, CD20, CD68, PD1, CXCL13, GAA^+^, PD1^+^CD3^+^, PD1^+^CXCL13^+^, GAA^+^PD1^+^, GAA^+^CD3^+^, GAA^+^CD20^+^, GAA^+^CD68^+^, GAA^+^CXCL13^+^, GAA^+^CD3^+^CXCL13^+^, GAA^+^CD3^+^PD1^+^, and CD3^+^PD1^-^CXCL13^+^ cells. The numbers and percentages of these cell populations were calculated.

### 2.8 Statistical analysis

Clinical responses were evaluated according to the 2014 Lugano classification. The overall response rate (ORR) to first-line therapy was defined as the proportion of patients achieving complete or partial response. OS was defined as the interval from diagnosis to death from any cause or last follow-up. Survival curves were estimated using the Kaplan–Meier method. Variables with P < 0.05 in univariate analyses were included in multivariate Cox proportional hazards regression models to identify independent prognostic factors. The proportional hazards assumption was assessed using Schoenfeld residuals. Correlations between variables were assessed using Spearman’s rank correlation test. All statistical analyses were two-sided, and a P-value < 0.05 was considered statistically significant. Analyses were performed using R software.

## 3 Results

### 3.1 Patient characteristics

A total of 39 patients with AITL were enrolled in this study, and their baseline characteristics are summarized in **Table 1**. All patients received CHOP (cyclophosphamide, doxorubicin, vincristine, and prednisone) or CHOP-like regimens as first-line therapy. The ORR to first-line treatment was 69.2% (27/39), including 20 CR and 7 PR. After a median follow-up of 65.3 months, the 5-year OS rate was 53.0%. To comprehensively characterize the molecular features of AITL, transcriptomic sequencing was performed on all 39 AITL specimens, among which 20 cases underwent matched proteomic profiling. In addition, proteomic sequencing was conducted on FFPE specimens from 5 cases of RLH, which served as disease controls.

**Table 1:** Baseline characteristic of 39 AITL patients.

Characteristics	n (%)
Age, median(range)	62 (37-81)
Sex(M/F)	1.6:1
Ann arbor stage	
I-II	2(5.1%)
III-IV	37(94.9%)
LDH	
Elevated	20(51.3%)
Normal	17(43.6%)
ECOG PS	
0-1	31(79.5%)
> 1	6(15.4%)
B symptom	
Yes	18(46.2%)
No	20(51.3%)
Extranodal, n (%)	
>1	10(25.6%)
<= 1	28(71.8%)
Bone involvment	7(17.9%)
IPI	
High risk	5(12.8%)
Medium-high risk	17(43.6%)
Low-medium risk	8(20.5%)
Low-risk	7(17.9%)

### 3.2 GAA upregulation predicts poor survival in AITL

**Figure 1:**
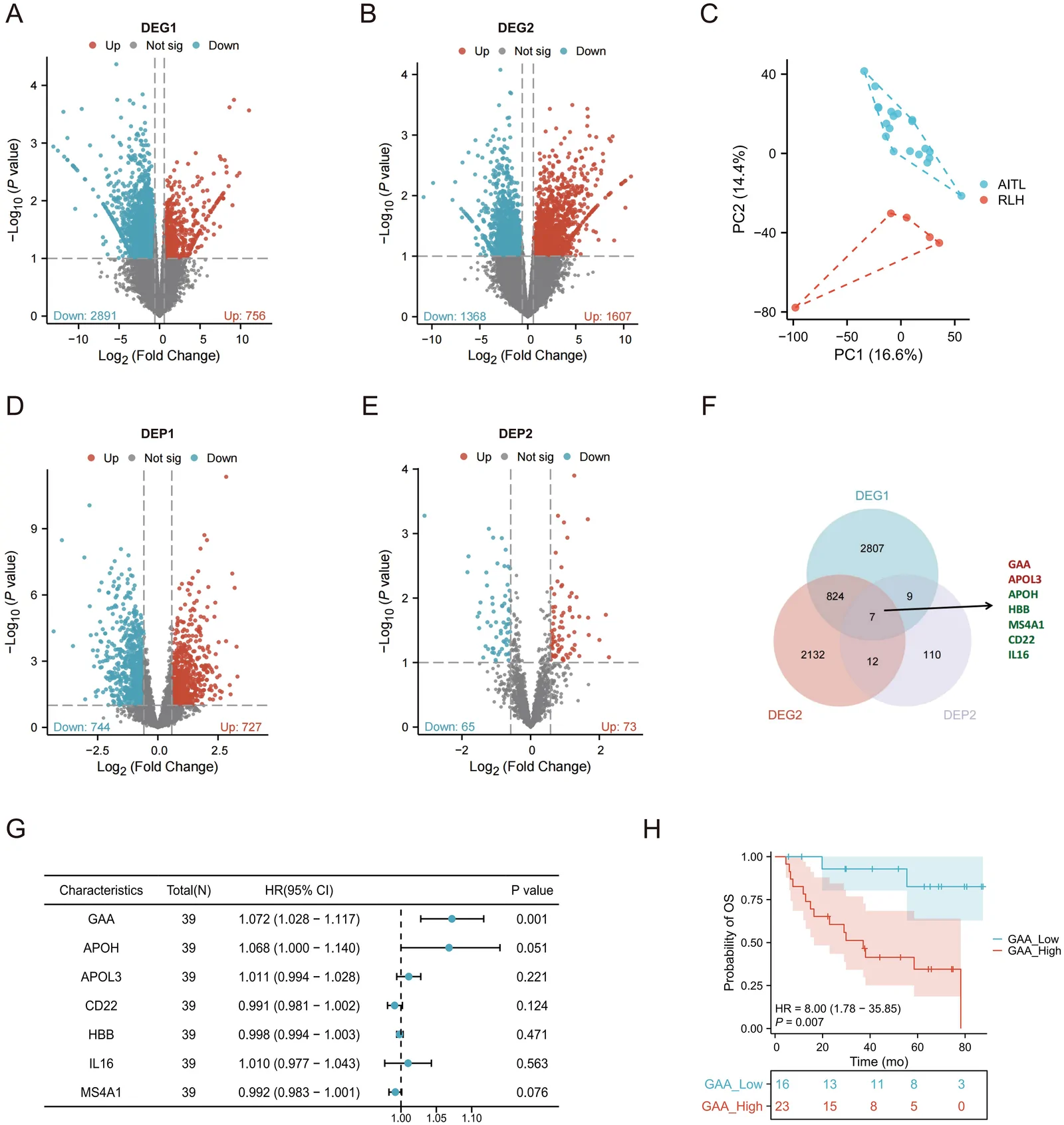
Multi-omics differential analysis identifies key biomarkers associated with clinical outcomes. **(A, B)** Volcano plots showing differentially expressed genes (DEGs) stratified by ORR in 39 AITL patients (DEG1) and 20 AITL patients (DEG2). **(C)** Principal component analysis (PCA) demonstrating clear separation between AITL (blue) and RLH (red) samples. **(D, E)** Volcano plots displaying differentially expressed proteins (DEPs) between AITL and RLH (DEP1), and DEPs stratified by ORR within the AITL cohort from DEP1 (DEP2). **(F)** Venn diagram illustrating the overlap of differentially expressed genes/proteins across DEG1, DEG2, and DEP2 datasets. **(G)** Forest plot of HR with 95% CI for seven candidate biomarkers. **(H)** Kaplan-Meier curves comparing survival by GAA expression levels.

To identify molecular features associated with treatment response in AITL, a multi-omics analytical framework was applied, as outlined in Figure Graphical Abstract. Differential gene expression analysis based on ORR status was first performed across the entire cohort of 39 AITL patients (DEG1), using thresholds of |log_2_FC| > 0.58 and nominal P < 0.1 (**Figure 1A**). False discovery rate (FDR)–adjusted P values were calculated using the Benjamini–Hochberg method and are provided in **Supplementary Table 2**. Given the limited sample size and the exploratory nature of this study, a nominal P-value threshold was applied for initial screening. This analysis identified 3,647 differentially expressed genes, including 756 up-regulated and 2,891 down-regulated genes in non-responders. To ensure consistency for integrated analyses, differential expression analysis was repeated in the subset of 20 patients with matched proteomic data (DEG2), yielding 2,975 differentially expressed genes (1,607 up-regulated and 1,368 down-regulated; **Figure 1B**; **Supplementary Table 3**).

The proteomic landscape of AITL was then characterized. In total, 5,508 proteins were quantified. Principal component analysis (PCA) revealed a clear separation between AITL and RLH samples, indicating distinct proteomic profiles (**Figure 1C**). Comparative analysis of baseline tumor proteomes from 20 AITL patients and 5 RLH controls identified 1,471 differentially abundant proteins (DEP1; |log_2_FC| > 0.58 and nominal P < 0.1; **Figure 1D**; **Supplementary Table 4**). To further identify proteins associated with treatment response, differential analysis based on ORR was performed within the AITL cohort, resulting in 138 response-related proteins (73 up-regulated and 65 down-regulated; DEP2; **Figure 1E**; **Supplementary Table 5**).

To identify molecules consistently associated with treatment response across transcriptomic and proteomic layers, three datasets (DEG1, DEG2, and DEP2) were integrated. Only molecules showing concordant directional changes across datasets were retained. Venn diagram analysis revealed seven overlapping candidates (**Figure 1F**). Among these, GAA and APOL3 were up-regulated in non-responders, whereas the remaining five molecules were down-regulated. These candidates represent integratively identified molecular features potentially associated with therapeutic response in AITL.

The prognostic relevance of the seven overlapping candidates was subsequently evaluated at the transcriptomic level. A total of 39 patients were included in the survival analysis, with 17 observed events. Among these seven candidates, only GAA expression was significantly associated with overall survival. Univariate Cox regression analysis demonstrated that higher GAA expression was significantly associated with inferior OS (hazard ratio [HR] = 1.072, 95% confidence interval [CI]: 1.028–1.117, P = 0.001; **Figure 1G**). The proportional hazards assumption was tested using Schoenfeld residuals, and no significant violation was observed for GAA (P = 0.30) or in the global test (P = 0.30). The optimal cutoff value for GAA expression was determined using X-tile software, and patients were stratified into GAA-high and GAA-low groups accordingly. Kaplan–Meier analysis showed that patients in the GAA-high group had significantly worse OS compared with those in the GAA-low group (HR = 8.00, 95% CI: 1.78–35.85, P = 0.007; **Figure 1H**). Sensitivity analysis using median-based stratification demonstrated a consistent trend toward worse OS in the GAA-high group, although statistical significance was not reached (HR = 2.29, 95% CI: 0.84–6.27, P = 0.107). The 5-year OS rates were 34.5% in the GAA-high group and 82.5% in the GAA-low group. Variables including gender, age, stage, LDH level, ECOG performance status, extranodal involvement (>1 site), B symptoms and IPI score were first evaluated by univariate Cox regression analysis. Older age was significantly associated with poorer OS. To account for established clinical prognostic factors, IPI score and GAA expression were incorporated into the multivariate Cox regression model. After adjustment, GAA expression remained significantly associated with OS (**Table 2**), suggesting that GAA may serve as a potential independent prognostic factor in this cohort.

**Table 2:** Univariate and multivariate analysis of prognostic factors for predicting the OS of AITL.

Characteristics	Total (N)	Univariate analysis		Multivariate analysis	
		Hazard ratio (95% CI)	P value	Hazard ratio (95% CI)	P value
Gender	39				
Male	24	Reference			
Female	15	1.339 (0.483 - 3.709)	0.575		
Age	39				
<=60y	15	Reference			
>60y	24	4.219 (1.193 - 14.921)	0.025		
Stage	39				
Stage I-II	2	Reference			
Stage III-IV	37	1.186 (0.156 - 9.027)	0.869		
LDH	37				
Normal	17	Reference			
Elevated	20	1.018 (0.381 - 2.718)	0.972		
ECOG PS	37				
0-1	31	Reference			
>1	6	1.409 (0.458 - 4.334)	0.550		
Extranodal>1	38				
No	28	Reference			
Yes	10	1.308 (0.412 - 4.151)	0.649		
B symptom	38				
No	20	Reference			
Yes	18	1.630 (0.619 - 4.294)	0.323		
IPI	37				
Low-risk	7	Reference		Reference	
Low-medium risk	8	1.005 (0.141 - 7.174)	0.996	1.146 (0.160 - 8.219)	0.892
Medium-high risk	17	2.835 (0.604 - 13.297)	0.186	1.888 (0.393 - 9.071)	0.428
High risk	5	3.956 (0.648 - 24.148)	0.136	2.238 (0.362 - 13.820)	0.386
GAA	39				
Low	16	Reference		Reference	
High	23	7.998 (1.784 - 35.847)	0.007	6.611 (1.409 - 31.032)	0.017

### 3.3 GAA expression is associated with CD8^+^ T-cell exhaustion

**Figure 2:**
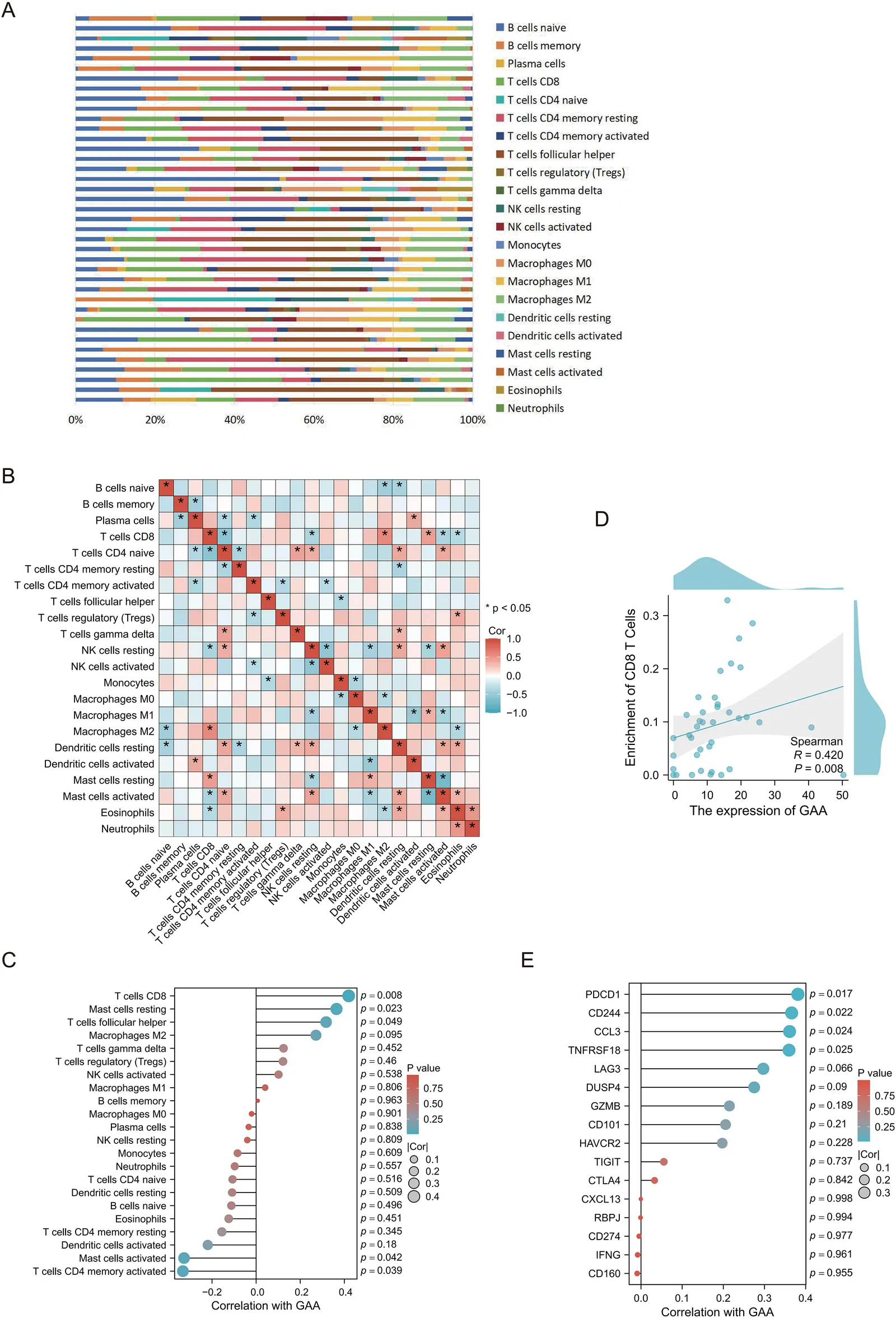
Immune landscape characterization and its associated with GAA expression. **(A)** Stacked bar chart depicting the relative proportions of 22 immune cell subtypes across individual samples, estimated by CIBERSORT deconvolution algorithm. **(B)** Correlation heatmap showing pairwise Spearman correlations among the 22 immune cell subtypes. **(C)** Forest plot displaying Spearman correlation coefficients between GAA expression and immune cell populations. **(D)** Scatter plot showing the positive correlation between GAA expression and CD8+ T cell infiltration. **(E)** Forest plot illustrating Spearman correlation coefficients between GAA expression and immune checkpoint molecules.

To further characterize the immune microenvironment in AITL, RNA-seq data from all samples were analyzed to estimate immune cell infiltration. The relative abundance of tumor-infiltrating immune cell populations, inferred using the CIBERSORT algorithm, is shown in **Figure 2A**. T follicular helper (Tfh) cells represented the most abundant immune subset, followed by naïve B cells, resting CD4^+^ memory T cells, and CD8^+^ T cells. Correlations among tumor-infiltrating immune cell populations are summarized in **Figure 2B**.

The relationship between GAA expression and immune cell infiltration was then examined. GAA expression showed a positive correlation with the abundance of CD8^+^ T cells (R = 0.420, P = 0.008; **Figures 2C, D**). Given this association, correlations between GAA expression and markers of T-cell exhaustion were further assessed. GAA expression demonstrated significant positive correlations with established exhaustion-related genes, including PDCD1 (PD-1), CD244, and CCL3 (**Figure 2E**).

To validate these findings, an independent cohort of 52 AITL cases from the publicly available dataset reported by Iqbal et al. (GSE19069 and GSE58445) was analyzed. Consistent with the discovery cohort, GAA expression was positively correlated with CD8^+^ T-cell infiltration in the validation dataset (**Supplementary Figures 1A, B**). In addition, GAA expression was broadly associated with an exhausted T-cell phenotype, as indicated by increased expression of exhaustion markers such as HAVCR2, LAG3, and CCL3 (**Supplementary Figure 1C**).

### 3.4 Functional enrichment reveals pro-invasive and metabolic alterations associated with GAA expression

**Figure 3:**
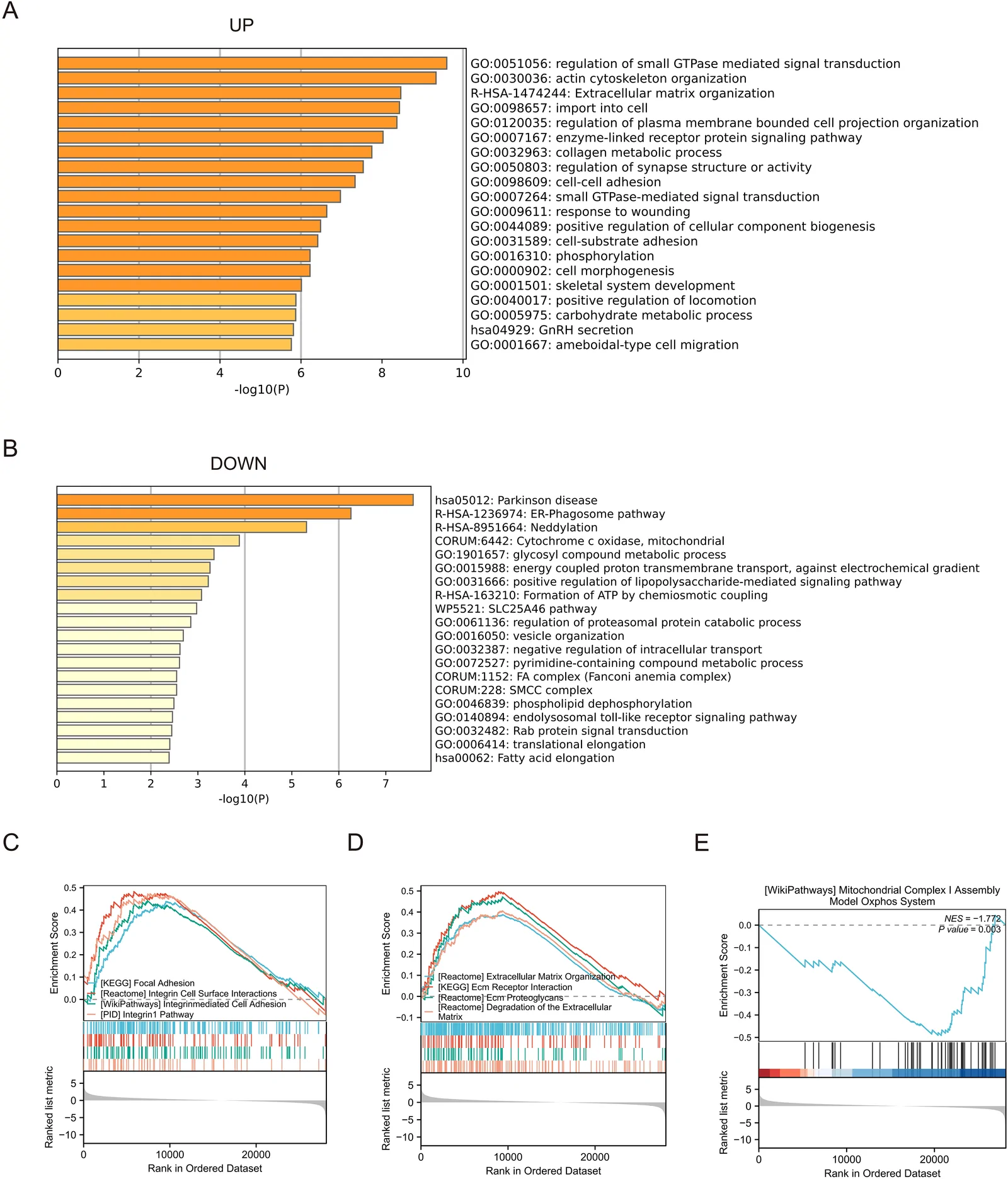
Functional enrichment analysis associated with GAA expression. **(A, B)** Metascape pathway analysis of genes positively correlated [**(A)**, UP] and negatively correlated [**(B)**, DOWN] with GAA expression. **(C–E)** Gene Set Enrichment Analysis (GSEA) plots showing enrichment of representative pathways associated with GAA expression.

To explore the functional implications of GAA-associated transcriptomic changes, differential gene expression analysis between GAA-high and GAA-low groups was followed by Metascape pathway enrichment analysis. Genes up-regulated in the GAA-high group were primarily enriched in pathways related to glycogen and carbohydrate metabolism, including carbohydrate metabolic processes and glucose phosphorylation, as well as pathways involved in cell adhesion, motility, and invasion, such as cell–cell adhesion, cell–substrate adhesion, and positive regulation of locomotion (**Figure 3A**). In contrast, down-regulated genes were significantly enriched in pathways related to mitochondrial function and fatty acid metabolism, including energy-coupled proton transmembrane transport, fatty acid elongation, and phospholipid dephosphorylation (**Figure 3B**).

Gene set enrichment analysis (GSEA) further confirmed these findings. The GAA-high group exhibited enrichment of pathways related to cell adhesion, migration, invasion, and extracellular matrix (ECM) remodeling (**Figures 3C, D**). Conversely, several immune-related and metabolic pathways were suppressed, including B-cell receptor (BCR) signaling and mitochondrial oxidative phosphorylation (OXPHOS) (**Figure 3E**). The suppression of OXPHOS was consistent with a metabolic shift toward glycolytic metabolism in GAA-high tumors.

### 3.5 IHC and mIF validation of GAA expression

**Figure 4:**
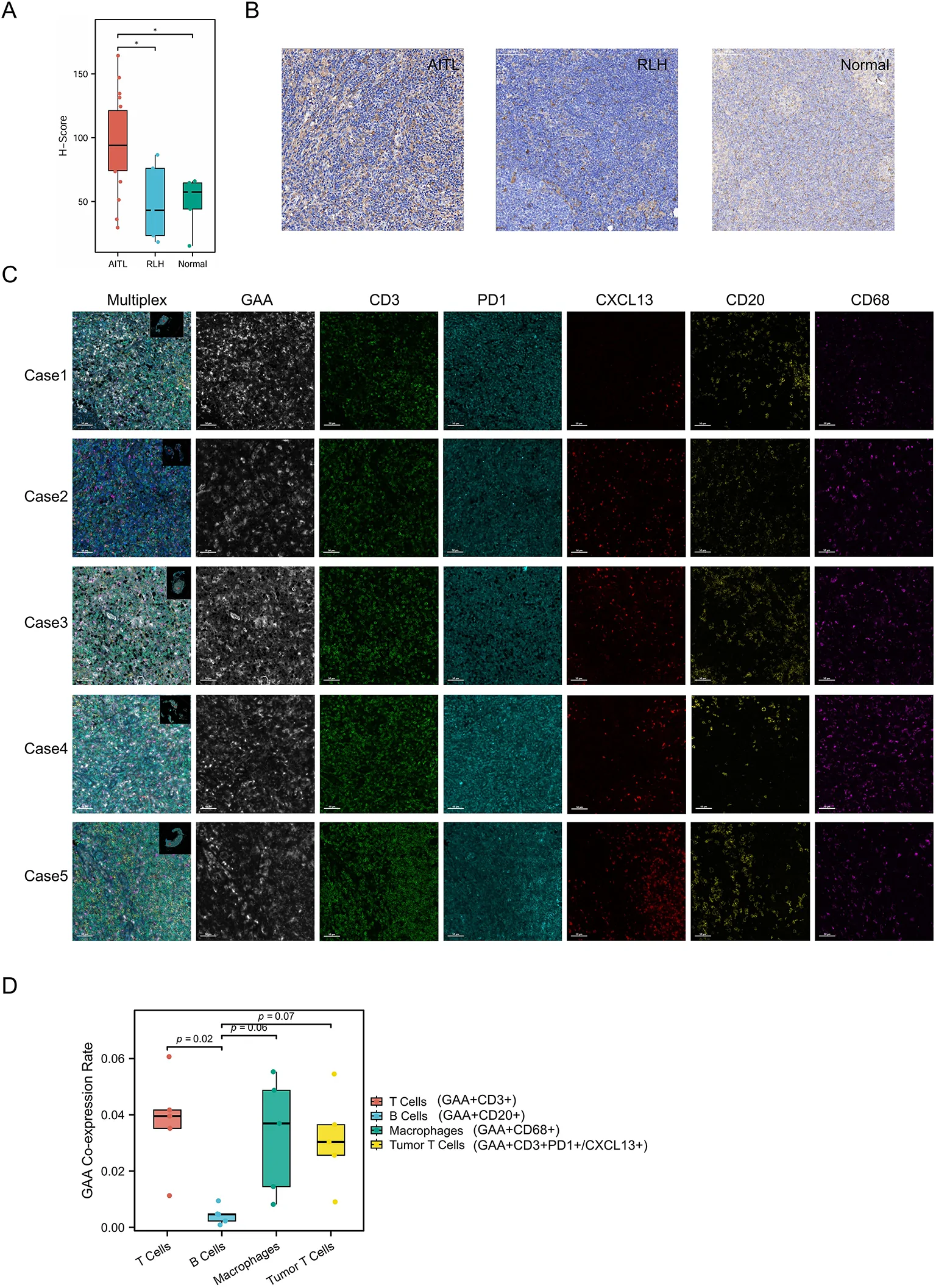
GAA expression pattern in AITL and its tumor microenvironment. **(A)** Box-and-scatter plot showing H-Score of GAA expression in AITL, RLH, and normal lymphoid tissues. **(B)** Representative immunohistochemical staining of GAA in AITL, RLH, and normal tissues. **(C)** The mIF staining images of 5 AITL samples. **(D)** Co-expression patterns of GAA and markers of T cells, B cells, macrophages, and tumor T cells.

To validate the elevated GAA expression observed in the transcriptomic and proteomic analyses, immunohistochemical staining was performed on FFPE tissues from 18 patients with AITL, as well as from 5 cases of RLH and 5 normal lymph nodes. Quantitative evaluation using H-scores demonstrated that GAA expression was significantly higher in AITL compared with RLH and normal lymphoid tissues, with median H-scores of 94, 43, and 57, respectively (**Figure 4A**). Representative immunohistochemical staining images are shown in **Figure 4B**.

To further characterize the cellular distribution of GAA within the tumor microenvironment, mIF was performed on 5 representative AITL cases (**Figure 4C**). GAA co-expression was predominantly observed in macrophages (CD68+) and T cells (CD3+), with the T-cell compartment mainly composed of tumor T cells (CD3+PD1+/CXCL13+) (**Figure 4D**; **Supplementary Table 6**).

## 4 Discussion

In this study, we conducted an integrated transcriptomic and proteomic analysis of AITL to explore molecular features associated with treatment response and clinical outcome. We identified GAA as a prognostically relevant molecule, with elevated expression consistently associated with inferior overall survival. Notably, increased GAA expression was accompanied by higher CD8^+^ T-cell infiltration while simultaneously correlating with markers of T-cell exhaustion. Together with the observed enrichment of metabolic pathways, these findings suggest that GAA-associated metabolic alterations may contribute to immune dysfunction within the AITL tumor microenvironment.

GAA is a lysosomal enzyme responsible for glycogen degradation into glucose. Although loss of GAA function is well characterized in Pompe disease, its role in cancer biology, particularly in hematologic malignancies, has received limited attention. Epidemiological studies have suggested that alpha-glucosidase inhibitors may reduce cancer incidence in patients with diabetes (14, 15), and experimental studies in solid tumors have shown that GAA suppression can enhance chemosensitivity and promote apoptosis through modulation of lysosomal and metabolic signaling pathways (16). In the present study, elevated GAA expression independently predicted poor overall survival after adjustment for established clinical prognostic factors, supporting a potential role for glycogen metabolism in AITL progression. This observation is in line with emerging evidence that altered glycogen utilization may provide metabolic flexibility and survival advantages to cancer cells under stress conditions (11, 17).

An important observation in our study is the association between high GAA expression, increased CD8^+^ T-cell infiltration, and the concurrent upregulation of exhaustion markers. While CD8^+^ T-cell infiltration is generally considered a favorable prognostic indicator in many malignancies, prior studies in AITL have reported the opposite association, linking increased CD8^+^ T-cell abundance to inferior outcomes. These findings suggest that CD8^+^ T cells in AITL may be functionally impaired rather than protective (18–21). Our data extend these observations by demonstrating that tumors with high GAA expression exhibit transcriptional features consistent with CD8^+^ T-cell exhaustion, providing a potential explanation for this paradox. This pattern resembles the “inflamed but dysfunctional” immune phenotype described in other tumor types, in which immune cell infiltration does not translate into effective antitumor activity (22).

The association between GAA upregulation and immune exhaustion may reflect tumor-driven metabolic reprogramming within the AITL microenvironment. Enrichment analyses indicate that GAA-high tumors exhibit transcriptional signatures suggestive of increased glycolytic pathway activity accompanied by reduced oxidative phosphorylation-related gene expression, consistent with a transcriptional profile compatible with aerobic glycolysis. Increased glycolytic flux has been associated with heightened glucose consumption and lactate accumulation, thereby creating a metabolically restrictive environment for effector immune cells. Previous studies have shown that tumor-derived lactate suppresses T-cell and NK-cell function through multiple mechanisms, including acidification of the tumor microenvironment, inhibition of T-cell glycolysis via monocarboxylate transporter 1 (MCT1), and activation of immunosuppressive signaling pathways (23). In addition, lactate has been reported to promote immune checkpoint expression, such as PD-L1, through HIF-1α stabilization, potentially reinforcing immune suppression. In this context, the observed correlation between high GAA expression, increased CD8^+^ T-cell infiltration, and elevated exhaustion markers suggests that enhanced glycogen breakdown may contribute to the establishment of a metabolically unfavorable niche. Such metabolic conditions are likely to compromise effector T-cell proliferation and function, given the reliance of activated T cells on glycolysis for cytokine production and clonal expansion (12, 24, 25).

Beyond immune-related effects, GAA-high AITL samples showed enrichment of gene sets involved in cell adhesion, migration, and ECM remodeling, features that may contribute to the aggressive clinical behavior and unfavorable prognosis observed in these patients. A growing body of evidence suggests that metabolic reprogramming is tightly coupled to invasive and migratory phenotypes in cancer. In this context, metabolic enzymes may influence tumor progression not only through their canonical metabolic functions but also via non-metabolic roles in gene regulation, cell signaling, and cytoskeletal organization (26–28). Although non-metabolic functions of GAA have not been defined, increased glycogen catabolism may enhance the availability of glucose and glycolytic intermediates, thereby supporting the biosynthetic demands associated with ECM production and tissue remodeling. Moreover, the pro-invasive transcriptional features observed in GAA-high tumors may reflect adaptive responses to metabolic stress, as tumor cells under high energetic demand often acquire increased migratory capacity to access more favorable microenvironments - a phenomenon reported across multiple cancer types.

Several limitations of this study should be acknowledged. The relatively small sample size necessitates validation in larger, independent cohorts. Moreover, the correlative nature of our analyses precludes definitive conclusions regarding causality, and functional studies manipulating GAA expression will be required to clarify its mechanistic role in tumor cells and immune components of the microenvironment. Finally, bulk transcriptomic and proteomic profiling reflects averaged signals across heterogeneous cell populations; future single-cell approaches may provide more precise insights into cell-type-specific expression patterns and interactions. From a translational perspective, investigation of pharmacologic strategies targeting glycogen metabolism, potentially in combination with chemotherapy or immunomodulatory approaches, warrants further exploration.

## 5 Conclusion

In summary, this integrative multi-omics analysis identifies GAA as an candidate prognostic marker in AITL, with elevated expression associated with poor overall survival. High GAA expression is associated with transcriptional signatures suggestive of enhanced glycolysis and reduced oxidative phosphorylation, as well as with increased CD8^+^ T-cell infiltration accompanied by transcriptional features of immune exhaustion. These findings indicate that GAA expression correlates with metabolic pathway alterations and immune microenvironmental features in AITL, highlighting GAA as a candidate therapeutic vulnerability warranting further functional validation.

## Data Availability

The datasets presented in this study can be found in online repositories. The names of the repository/repositories and accession number(s) can be found in the article/**Supplementary Material**.
