# Supplementary material for: Multi-omics analysis identifies GAA as an independent poor prognostic biomarker and candidate therapeutic target in angioimmunoblastic T-cell lymphoma
Source: Front Immunol. 2026 Aug 11;17:1862168. doi: 10.3389/fimmu.2026.1862168 (PMC13503525; doi:10.3389/fimmu.2026.1862168)
Supplement: Supplementary file 1 [file Table2.docx]

**Supplementary materials**

**Supplementary Fig.1 Validation of GAA-associated immune landscape in an independent AITL cohort (GSE19069 and GSE58445).**

(A) Forest plot showing Spearman correlation coefficients between GAA expression and 22 immune cell populations estimated by CIBERSORT. (B) Scatter plot demonstrating the positive correlation between GAA expression and CD8+ T cell enrichment in the validation cohort. (C) Forest plot displaying Spearman correlation coefficients between GAA expression and immune checkpoint molecules in the validation cohort.
